# Graph Theoretical Methods and Workflows for Searching and Annotation of RNA Tertiary Base Motifs and Substructures

**DOI:** 10.3390/ijms22168553

**Published:** 2021-08-09

**Authors:** Reeki Emrizal, Hazrina Yusof Hamdani, Mohd Firdaus-Raih

**Affiliations:** 1 Department of Applied Physics, Faculty of Science and Technology, Universiti Kebangsaan Malaysia, UKM Bangi, Bangi 43600, Selangor, Malaysia; reeki@mfrlab.org; 2 Institute of Systems Biology, Universiti Kebangsaan Malaysia, UKM Bangi, Bangi 43600, Selangor, Malaysia; 3 Advanced Medical and Dental Institute, Universiti Sains Malaysia, Bertam, Kepala Batas 13200, Pulau Pinang, Malaysia

**Keywords:** RNA structure, 3D base motifs, base interaction networks, base clusters, graph theory, RNA structure annotation

## Abstract

The increasing number and complexity of structures containing RNA chains in the Protein Data Bank (PDB) have led to the need for automated structure annotation methods to replace or complement expert visual curation. This is especially true when searching for tertiary base motifs and substructures. Such base arrangements and motifs have diverse roles that range from contributions to structural stability to more direct involvement in the molecule’s functions, such as the sites for ligand binding and catalytic activity. We review the utility of computational approaches in annotating RNA tertiary base motifs in a dataset of PDB structures, particularly the use of graph theoretical algorithms that can search for such base motifs and annotate them or find and annotate clusters of hydrogen-bond-connected bases. We also demonstrate how such graph theoretical algorithms can be integrated into a workflow that allows for functional analysis and comparisons of base arrangements and sub-structures, such as those involved in ligand binding. The capacity to carry out such automatic curations has led to the discovery of novel motifs and can give new context to known motifs as well as enable the rapid compilation of RNA 3D motifs into a database.

## 1. Introduction

For decades, the deposition of RNA structure coordinate data in the central repository of biological macromolecular structures, the Protein Data Bank (PDB) [1], has lagged behind that of proteins. This is, in part, due to the long-held understanding of RNA’s role as only that of an intermediary molecule that bridges the storage capacity of DNA to the functionality of proteins, as dictated in the central dogma of molecular biology.

**Figure 1:**
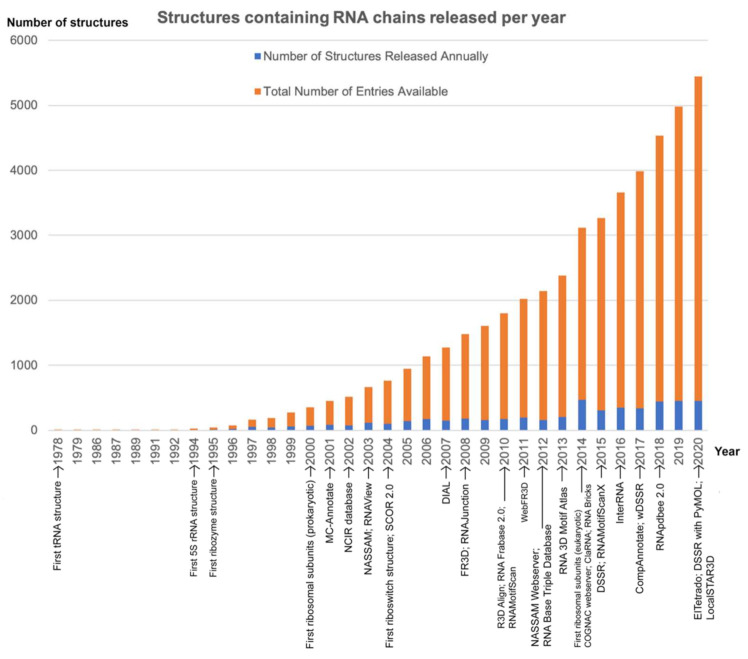
Availability of RNA-chain-containing structures in the Protein Data Bank, with annotations of significant structure submissions and publication of tools that processed or analyzed RNA structures available in the PDB [4,5,6,7,8,9,10,11,12,13,14,15,16,17,18,19,20,21,22,23,24,25,26,27,28,29,30,31,32,33,34,35,36,37].

Developments in the 1980s presented evidence that RNA itself is a directly functional molecule and not a mere relay of genetic information [2]. The discovery of catalytic RNAs has paved the way for the elucidation of various other functional roles of non-protein coding RNA. In the RNA world hypothesis, interactions of catalytic RNAs such as ribozymes with various coenzymes can expand the chemical range of its catalysis, thus making RNA-based metabolism feasible. Such ribozymes with novel chemistry could be discovered by investigating organisms living in isolated or extreme environments or employing bioinformatics-based structure and sequence homology approaches [3]. To better understand the atomic-level mechanisms for the functions of these RNA molecules, efforts have been made to crystallize them and acquire their molecular structure coordinates. Previous efforts at obtaining RNA structure coordinates have been limited to that of transfer RNA (tRNA) molecules. Among the first non-tRNA structures solved was for that of a helical fragment of *E. coli* 5S rRNA (PDB ID: 1ELH) [4], deposited in the PDB in 1994 (Figure 1).

The limited availability of RNA structure coordinate data and their lack of fold diversity compared to proteins prior to the year 2000 meant that there was a lack of need and urgency for the development of computational tools that could annotate and compare large numbers of RNA structures (Figure 1). Recent developments in protein structure prediction also revealed that methods utilizing deep learning approaches can result in highly accurate tertiary structure predictions [38]. Such a scenario could soon be true for the field of RNA structure modeling, thus resulting in a fast and marked increase in the volume of available RNA tertiary structures. Advancements in cryogenic electron microscopy (cryo-EM) technology have enabled large RNA–protein or RNA-only complexes to be solved, thus initiating an RNA structure renaissance; this has ignited interest on whether many RNA structures, other than those already known as drug targets, may also contain binding pockets that could be targeted for therapeutic applications [39,40]. The more complex structures also made clear that the arrangements of base–base interactions were much more diverse than the canonical interactions observed for DNA [41]. This, in turn, has led to efforts to annotate base interactions in available RNA structures and to identify the presence of tertiary base–base interaction motifs.

Early efforts at identifying such tertiary motifs in RNA structures relied on manual curation using molecular visualization software, and one notable early effort is the non-canonical interactions in the RNA database (NCIR) [10]. Such manual visual curation approaches were also used for the larger-sized ribosomal subunit structures, and one notable example resulting from such work was the discovery of A-minor motifs [42].

Manual visual curation is highly dependent on the expertise and observational acuity of the investigator and, thus, clearly restrictive, especially as the number of available RNA structures increase. The limitations of manual curation became more obvious with the availability of the complex structures of large ribosomal subunits [43,44]. This situation clearly highlighted the need for the development of automated approaches to annotate RNA structures. Such methods include those that classify structures based on the folding of the RNA backbone [45] as well as those that classify the tertiary arrangements of the bases [46].

As the diversity and volume of RNA structures available in the PDB increase, efficient computational tools that can process such coordinate data in a high-throughput manner to allow for the discovery of novel motifs and to annotate known arrangements will be needed. These tools would also have the added requirement of being able to compare the presence of base sub-structures in different structures, including the large and complex structures of the ribosomal assemblies. In this paper, we present methods and protocols to annotate known 3D base arrangements, identify novel motifs, and compare the presence of such tertiary arrangements in different RNA structures.

## 2. Algorithms for Annotating RNA 3D Base Arrangements

The PDB serves as the central repository of biological macromolecular structures, and, due to this, the algorithms we present and discuss will center around the capacity to process file formats that are available in the PDB, namely, the legacy *.pdb and the current *.cif formats. We then take into consideration the need to: (i) identify known types of base arrangements in the available PDB structures, and (ii) identify previously undescribed arrangements and classify novel motifs. To accomplish this, we present and discuss the utility of a computer program that can annotate 3D base arrangements in RNA structures and a computer program that can annotate networks of RNA base clusters that are inter-connected by hydrogen bonds. We further consider the requirement that regions containing sub-structures or motifs may need to be compared in different structures to allow for functional implications of atomic-level structural differences to be investigated and explored.

### 2.1. Comparison of Computational Approaches in Annotating RNA Base Motifs

There are numerous computational approaches currently available in the form of webservers, databases, or downloadable executables (Table 1) [6,7,8,9,10,11,12,13,14,15,17,19,20,21,22,23,24,25,26,28,29,30,31,33,34,35,36]. The variety of methods, inputs, and outputs that are offered by the currently available programs (Table 1) create a rather complementary RNA base motif analysis ecosystem [6,7,8,9,10,11,12,13,14,15,17,19,20,21,22,23,24,25,26,28,29,30,31,33,34,35,36]. Many of those approaches, such as NASSAM, COGNAC, MC-Annotate, R3D align, RNA-Bricks, RNAMotifsScanX, and LocalStar3D (Table 1) [9,18,22,26,28,30,35], employ graph theoretical algorithms. However, due to the different objectives of each program, the graph representations used also differ. The programs R3D align and LocalStar3D adopt graph-based alignments, where the nodes are the local base alignments and the edges connect the alignments [18,35]. RNA-Bricks adopts a reduced graph representation using 3D motifs such as loops, stems, or single strand terminals as the nodes and the nucleotide pairs, intra-molecular interactions, or crystallographic contacts as edges [28].

The MC-Annotate, NASSAM, and COGNAC programs are similar because they consider the ribonucleotide components as the graph’s nodes and the distances between the ribonucleotides as the edges of the graph [9,22,26]. RNAMotifScanX also utilizes graph-based alignments, where the nodes are the base residues and the edges are base interactions between those residues [30]. MC-Annotate employs a sub-graph isomorphism algorithm through the program MC-Search [9,22,26]. However, unlike MC-Annotate, the methods used by NASSAM and COGNAC webservers represent each ribonucleotide as two vectors that consist of pseudo-atoms as the nodes and pairwise atomic distances as the edges. This reduces the whole RNA structure to pseudo-atom vector arrangements that can be solved by the sub-graph isomorphism algorithm [22,26]. Introducing more nodes to represent each ribonucleotide can confer greater specificity to the motif searches performed by the NASSAM webserver. This has allowed for the annotation of motifs that are not identified by MC-Annotate; previously reported examples of this are base triples annotated by NASSAM in the structures with PDBIDs 3QIR and 3G78 that were not identified by MC-Annotate [22]. The NASSAM webserver also allows the identified base triple motifs to be filtered for more stable interactions (presence of at least two hydrogen bonds); it also allows an increase in search tolerance to extend motif search coverage [22].

RNApdbee 2.0 and El Tetrado enable the analysis of quadruplexes that could be useful for studying telomeric motifs that are often associated with cancer and neurodegenerative diseases [34,36]. Motif analysis on ribosomal structures that are of low resolution could benefit from the method employed by CompAnnotate [32]. For tertiary structure analyses that require a wider range of motif annotations, users can opt for WebFR3D, RNA Frabase 2.0, and NASSAM [9,18,22,26,28,30,35]. The primary focus of this paper will be on a suite of graph theoretical algorithms with web browser interfaces because such accessibility could allow for a wider user base.

### 2.2. Annotation of Tertiary Base Arrangement Using the NASSAM Computer Program

**Figure 2:**
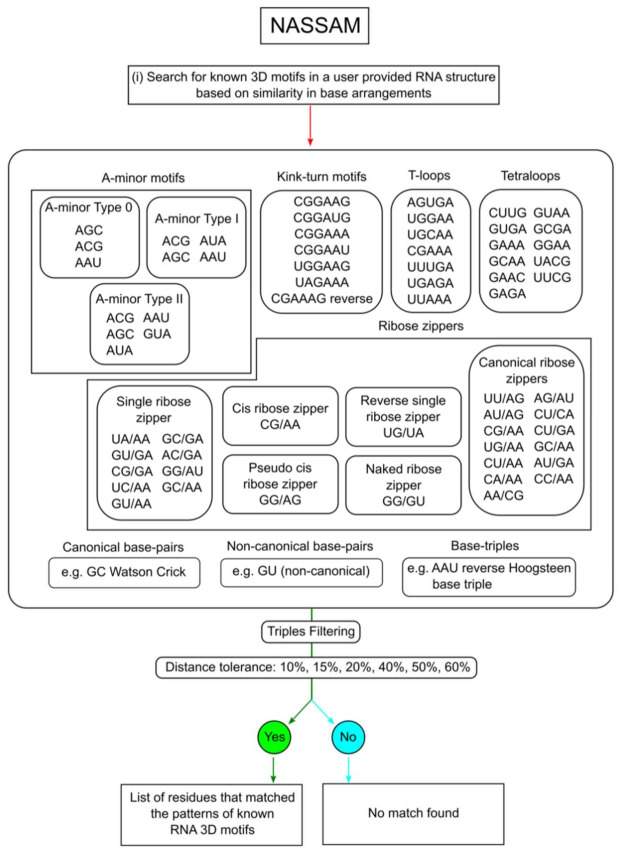
A flowchart outlining the search process for base arrangements in RNA structures that exhibit similar 3D geometry with known RNA base arrangements, performed by the NASSAM webserver.

The NASSAM (Nucleic Acids Search for Sub-structure And Motifs) computer program employs the Ullmann sub-graph isomorphism algorithm to identify similar base arrangements in PDB structures containing RNA chains [11]. In its original implementation, the program required an input base arrangement and searched a structural database for occurrences that matched the query and, thus, was very similar to its counterpart for amino acid side chain similarity searching, ASSAM [47]. The program has since been implemented as a webserver and differs in operation from the original program reported by Harrison et al. in accepting a whole RNA chain containing PDB structure as input to search against a database of known RNA base arrangements (http://mfrlab.org/grafss/nassam/ accessed on 1 August 2021) (Figure 2) [22].

**Figure 3:**
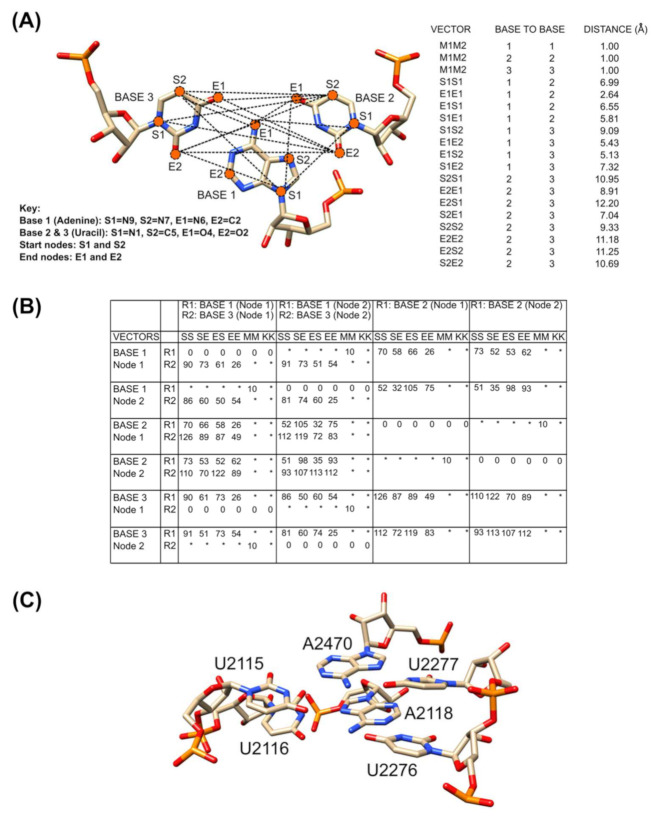
(**A**) The distances between the pseudo-atoms (left panel) that represent the 3D arrangement of the base triple provided are extracted (right panel) and (**B**) converted into matrices to be computed and solved as a sub-graph isomorphism problem. As an example on how to read the matrices (Figure 3B), E1E1 distance between Base 1 and Base 2 that is 2.64 Å (right panel of Figure 3A) is marked as 26 (distance ×10) under the EE column of Base 1 (Node 1) and the R1 row of Base 2 (Node 1) in the matrices. The distance of 0 Å is for the instances where the nodes map into itself and * is for the instances where the distance is undefined. (**C**) A-stacked UAU base triple found in domain V of the 23S subunit of prokaryotic rRNA.

In NASSAM, RNA bases are represented as pseudo-atom vectors (Figure 3A). The coordinates of the query structure and the motif-containing search database are both converted into their pseudo-atom vector representations. The 3D geometry that represents the spatial arrangements of the bases to each other are represented as graphs (matrices), and the searches are solved as sub-graph isomorphism problems (Figure 3B). A query structure can be submitted in the form of a coordinate file, and the structure is then searched against a database that includes both previously recorded motifs and hypothetical base arrangements. The result of a NASSAM search is an annotation of the 3D arrangements present in the query RNA-chain-containing structure. One limitation of such an annotation system is that it is dependent on the arrangement already being known or hypothetically conceived and present in the database provided.

### 2.3. Annotation of Hydrogen-Bond-Connected Base Clusters Using the COGNAC Computer Program

**Figure 4:**
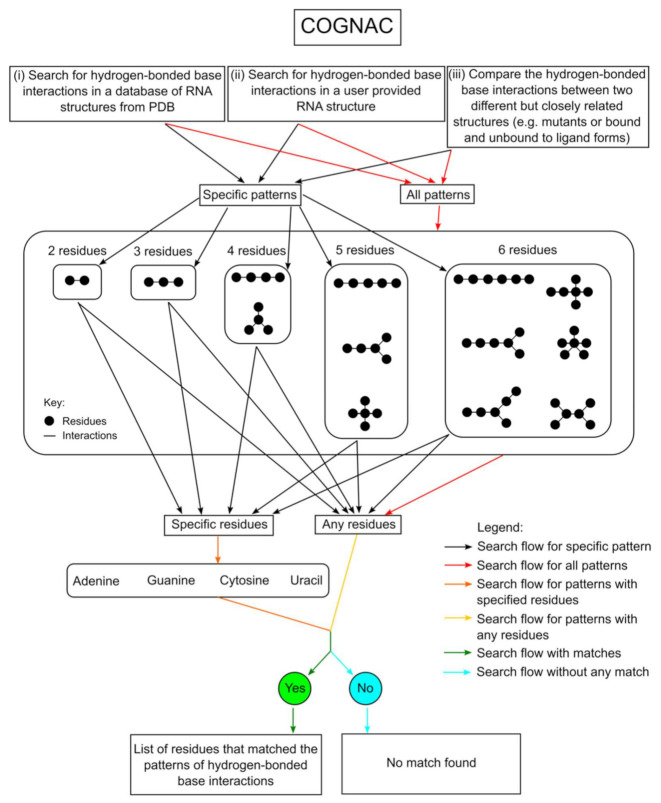
A flowchart that describes the search for hydrogen-bonded base interactions in RNA structures, performed by the COGNAC webserver.

The NASSAM program requires prior knowledge of a previously described base arrangement and, thus, may not be very useful for the discovery of novel base arrangements. In order to complement the annotation capabilities of NASSAM, the COGNAC (COnnection tables Graphs for Nucleic ACids) computer program was developed to allow for a systematic approach to identify novel 3D base motifs. The COGNAC computer program is also accessible via a web interface (http://mfrlab.org/grafss/cognac/ accessed on 1 August 2021) [26] (Figure 4).

**Figure 5:**
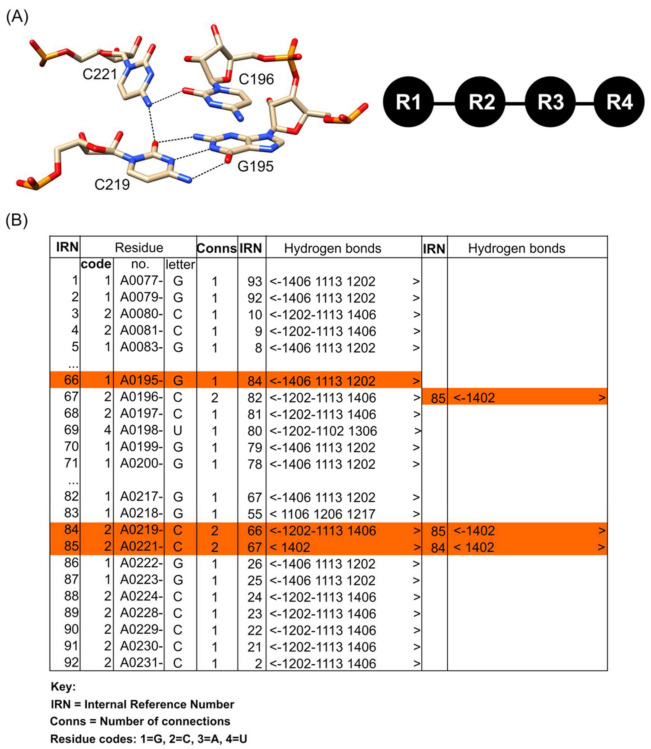
(**A**) A base quadruple inter-connected by hydrogen bonds (left panel), represented as a tree graph (right panel). (**B**) Hydrogen bonds computed and arranged as a connection table, with the hydrogen bonds involved in the four-base network highlighted in orange.

Structures to be annotated by COGNAC are first processed by an internal program that calculates the possible hydrogen bonds between the bases as a connection table. Each hydrogen-bond-connected base network from the connection table is considered a tree graph, with the bases as the nodes and the hydrogen bond(s) as the edges (Figure 5). The structures in the search database are similarly processed. The COGNAC program then solves a sub-graph isomorphism problem to match the query graph as a sub-graph within the larger graphs representing the whole RNA structure.

## 3. Workflows for Annotating RNA 3D Base Arrangements

Here, we discuss two approaches for annotating 3D base arrangements and motifs in PDB structures containing RNA chains. The first involves the annotation of tertiary base arrangements using the NASSAM computer program based on the spatial geometry of the bases. The user submits an RNA structure coordinate file to be annotated, and the program returns a list of tertiary arrangements that match those in a database of previously reported RNA base motifs as well as hypothetically computed base arrangements (Figure 3). The second approach annotates tertiary base arrangements based on them containing an unbroken network of hydrogen bonds connecting the bases in the pattern (Figure 5). This allows for tightly connected clusters of bases that could be of structural and/or functional importance to be identified.

### 3.1. Searching for Novel RNA Base Motifs

There are several resources that contain information on RNA 3D motifs that have been discovered in actual RNA structures [23,24,31]. Using prior knowledge of what these base arrangements are, it is then possible for computer programs to search for them in other RNA structures. Such searches are clearly limited to the capacity to annotate for known arrangements of motifs only. However, using a dataset of theoretical base arrangements as queries for such searches, it was demonstrated that novel arrangements that are present in the currently available dataset of RNA structures could be discovered [48].

Nevertheless, the combinations of possible base arrangements are evidently more diverse and complex than the set of hypothetical arrangements reported. Therefore, it is clear that such a search capacity does not address the problem of how novel motifs or base arrangements can be discovered. In many cases, the discovery of a novel base motif would be due to an individual investigator reporting a previously undescribed base arrangement and finding repeat occurrences of the arrangement in other structures. This limits the rate of discovering novel motifs even though the motifs are present and awaiting discovery in a dataset of already available structures.

Searching for novel motifs using a known arrangement is possible. In the case of the NASSAM computer program, a search using a known arrangement as a query can be set to a very high tolerance, which will result in the retrieval of highly divergent arrangements from the original queries. However, due to the high computational resource and subsequent filtering requirements, such a search option is not made available via the webserver interface.

An alternative approach that was explored involved the use of base interaction networks. This was based on the premise that if a cluster of bases is inter-connected by hydrogen bonds, then there might be a specific functional or structural requirement for those bases to be in such close proximity. Therefore, analysis of base interaction networks to identify similarities in the hydrogen-bonding network pattern may identify patterns that are repeated in different structures, thus allowing them to be classified as a motif. In cases where such arrangements have not been reported, the discovery of a novel base arrangement motif will have been achieved. This particular approach utilizes the COGNAC computer program [26,49].

### 3.2. Application of 3D Base Arrangement Comparisons to Identify Known Motifs in a Novel Context

A novel RNA base motif need not be limited to only previously unreported arrangements. A known base arrangement can also have a novel context that, in itself, can also be considered as a novel motif. As an example, the NASSAM computer program was previously used to search for 942 hypothetical base triple arrangements in available RNA structures. Among the arrangements that were searched for were base triples composed of UAU [48]. The NASSAM searches were indeed able to identify various UAU base triples. One particular arrangement, a UAU Hoogsten Watson-Crick base triple, was found to be stacked together with exactly the same triple arrangement in domain V of the ribosomal subunits (Figure 3C) [48]. The presence of this highly conserved interaction at a junction structure implies its potentially important role in maintaining the structural integrity of the domain in the ribosomal subunits.

### 3.3. Application of 3D Base Arrangement Searching to Identify Functional Sites

**Figure 6:**
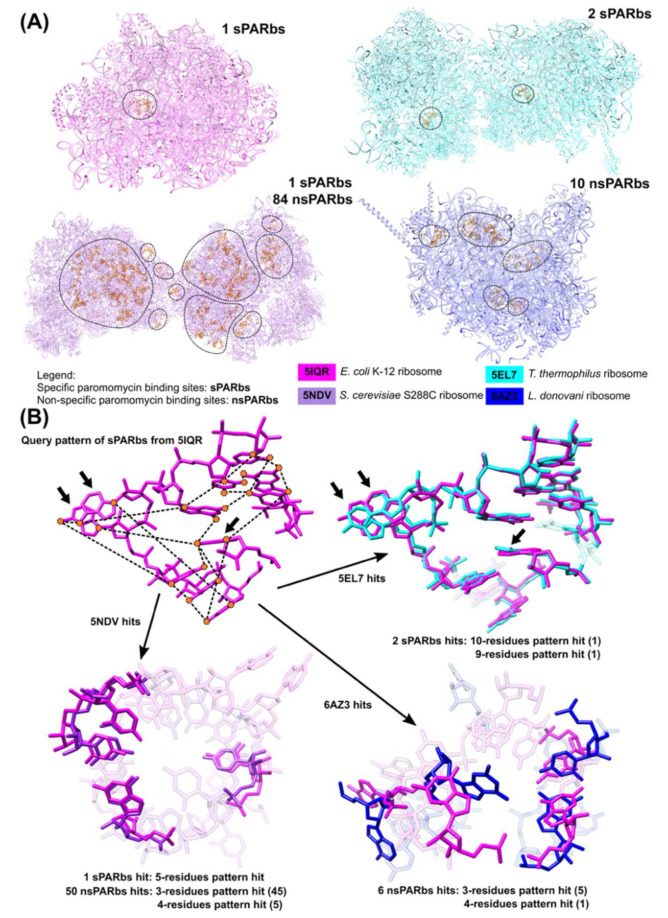
Identification of functional (paromomycin binding) sites in RNA structures from the PDB by NASSAM. (**A**) The regions on RNA structures (PDB ID: 5IQR, 5EL7, 5NDV, and 6AZ3) that contain the extracted binding sites are enclosed within black dashed lines and the binding sites are represented as orange sticks. (**B**) The specific paromomycin binding site from 5IQR that is identified by the presence of an AA-A motif (black arrows) is used as a query pattern in the NASSAM search. In the query pattern, the pseudo-atoms of bases are denoted as graph nodes (orange circles) and the distances between pseudo-atoms are denoted as graph edges (black dashed lines). The query pattern is used by the NASSAM algorithm to search for sites that exhibit similar base arrangements (hits) in other RNA structures.

The algorithms and workflows employed by the NASSAM [22] and COGNAC [26] programs could also be adopted for the comparison of 3D base arrangements to identify functional sites. A functional site in this instance is defined as an RNA sub-structure that is known to interact with ligands such as drug molecules, ions, amino acids, nucleotides, and metabolites, where such interactions can modulate the biological activity of the RNA molecule [46]. To demonstrate this utility, a dataset of four structures of ribosomal complexes that bind paromomycins from prokaryotes (*Escherichia coli* (PDB ID: 5IQR) and *Thermus thermophilus* (PDB ID: 5EL7)) and eukaryotes (*Saccharomyces cerevisiae* (PDB ID: 5NDV) and *Leishmania donovani* (PDB ID: 6AZ3)) that were solved to at least 3.30 Å resolution was retrieved from the PDB [50,51,52,53]. The Representative Sets of RNA 3D Structures database (version 3.189) from the BGSU RNA site was referenced to identify redundant structures [54]. Out of the four structures, 6AZ3 is considered a representative structure. The other three structures (5NDV, 5IQR, and 5EL7) can also be represented by other structures. However, the representative structures used do not have paromomycins bound to them. Furthermore, since none of the four structures shared similar ribosomal representative structures, those structures can be considered non-redundant to each other [54]. The structures were processed using an automated program written using Python (version 3.8). As part of the requirement of downstream processes in the pipeline, the coordinates of the structures in *.cif format were modified into *.pdb format by the program by splitting the coordinates of groups of chains into separate files. Then, the base arrangements of RNA structures that are in contact with paromomycin (defined as being a distance of 4 Å or less between a nucleotide’s atom and a ligand’s atom) were extracted by the program. The regions that contain the extracted binding sites are shown on their respective structures (Figure 6A).

It has been reported that paromomycin is able to specifically bind at the internal loop of the 30S ribosomal subunit’s A-site. The specific binding pocket is characterized by the presence of an A-A base pair and a bulged adenine (prokaryotes) or guanine (eukaryotes) (top panels of Figure 6B) [55]. Hence, the extracted binding sites that fit the characteristics are classified as specific paromomycin binding sites (sPARbs); otherwise, they are classified as non-specific paromomycin binding sites (nsPARbs) (Figure 6A).

For the purpose of demonstrating the application of this workflow, only the patterns that describe the geometric relationships between pseudo-atoms of the bases for the sPARbs from 5IQR were generated by the program and used by the NASSAM algorithm [22] to search for similar tertiary base arrangements in other RNA structures (Figure 6B). Multiple patterns were generated for a base arrangement to consider all possible combinations of components (nucleotides) of the binding site. As an example, the 11-nucleotide sPARbs from 5IQR generated 1981 patterns for all possible combinations of the nucleotides down to 3-nucleotide combinations. The program interacts with the UCSF Chimera molecular visualization interface to compare the query (sPARbs from 5IQR) with the hits found by NASSAM using the least-squares superposition method [56] (Figure 6B).

The NASSAM searches were able to retrieve other sPARbs in other RNA structures (PDB ID: 5IQR, 5EL7, 5NDV), although there were differences in the components that make up the sPARbs in those structures. The sPARbs for PAR1691 of chain 1G from 5EL7 have ten similarly arranged bases (RMSD value: 0.730 Å) (Figure 6B) and the sPARbs for PAR1749 of chain 13 from 5EL7 contain nine similarly arranged bases (RMSD value: 0.723 Å); the sPARbs for PAR1905 of chain 2 from 5NDV consist of five similarly arranged bases (RMSD value: 1.398 Å) compared to the 11 nucleotides of the query pattern from the sPARbs for PAR1665 of chain 2 from 5IQR. This illustrates the capability of the program and workflow to find other similar base arrangements that share similar 3D geometry despite consisting of different combinations of base components from the query pattern. Due to the sequence-independent nature of the search, the similar base arrangements found by the NASSAM algorithm do not necessarily share a sequence motif but share a 3D geometric conservation that can still be considered a motif due to its recurrent nature among those base arrangements.

The nsPARbs for PAR3424 of chain 1 from 5NDV have four similarly arranged bases (RMSD value: 1.321 Å) (Figure 6B) while the nsPARbs for PAR1602 of chain 2 from 6AZ3 also contain four similarly arranged bases (RMSD value: 6.237 Å) (Figure 6B), although lacking the AA-A motif that is usually present in sPARbs (Figure 6B) [55]. Despite the overall misalignment between the queried sPARbs and both nsPARbs from 5NDV and 6AZ3 (Figure 6B), the conserved 3D geometry observed among those binding sites may have a role in maintaining the three-dimensional space required for ligand binding (Figure 6B). This demonstrates that a sub-structure search program such as NASSAM is able to identify sites in other RNA structures that may have a functional role, such as ligand-binding interfaces.

**Figure 7:**
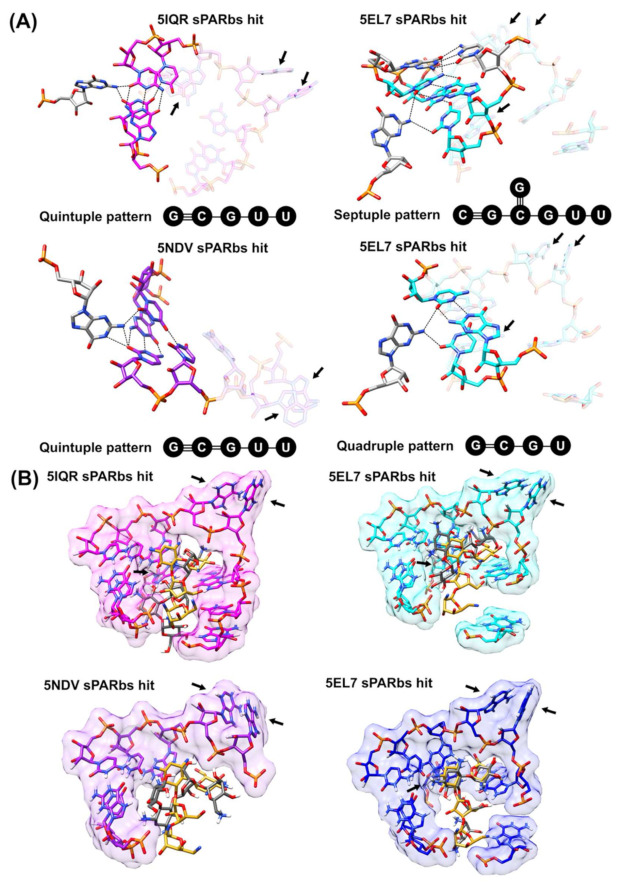
Annotation of functional (paromomycin-binding) sites in RNA structures from PDB by COGNAC. (**A**) The components of binding sites from RNA structures (PDB ID: 5IQR, 5EL7, and 5NDV) that involve in the networks of hydrogen-bonded base interactions are colored in magenta, cyan, and purple, respectively; otherwise, they are transparent. The nucleotides that are not components of binding sites but are involved in the interaction networks are colored in grey. The networks of hydrogen-bonded base interactions are represented by the dashed lines. (**B**) The surface renderings represent the possible binding pockets formed. The predicted binding pose (grey paromomycin) and the experimentally determined binding pose (gold paromomycin) are shown in those binding pockets. The hydrogen, nitrogen, oxygen, and phosphate atoms are colored in white, blue, red, and orange, respectively, on the stick structures in (**A**,**B**).

Networks of hydrogen-bonded interactions have been reported to be important for the stabilization and catalytic mechanism in the group I ribozyme active site [57]. Thus, the incorporation of the searching of unbroken networks of hydrogen-bonded base interactions by the COGNAC program [26] in the workflow can provide additional value to the annotation of sites and motifs by the NASSAM program [22]. The program interacts with the COGNAC program to identify whether any components that make up the similar base arrangements identified by the NASSAM algorithm are involved in a cluster of hydrogen-bonded base interactions. The COGNAC algorithm is able to identify higher-order hydrogen-bonded network patterns that involve components of sPARbs from 5IQR, 5EL7, and 5NDV (Figure 7A). The G-C and U-U base pairs from the sPARbs of 5IQR and 5NDV, together with a guanine that is in close proximity to those sPARbs, form a quintuple pattern.

The components of the two sPARbs from 5EL7 were also found to be involved in a network of hydrogen-bonded base interactions, although there was a variation in the patterns formed. One of the sPARbs exhibits a sextuple pattern that involves G-C and U-U base pairs from the sPARbs, together with a guanine and G-C base pair that is in close proximity to the sPARbs (Figure 7A). The other sPARbs contain a quadruple arrangement that involves a G-C base pair and a uracil from the sPARbs, together with a guanine that is in close proximity to the sPARbs (Figure 7A). These variations might have specific structural and functional implications; thus, the prediction of such networks of hydrogen-bonded base interactions can provide insights into the unique mechanisms associated with each different functional site.

Molecular docking was incorporated into the automated program workflow to assess the potential of the ligands to bind the sites identified by the NASSAM program [22]. The workflow uses AutoDockTools to prepare the ligands and receptors for docking by adding hydrogens and Gasteiger charges [58]. The prepared inputs are then passed to AutoDock Vina for the docking analysis [59] using default parameters: exhaustiveness value is eight, dimension of search space is 30 Å × 30 Å × 30 Å, and number of binding modes to be generated is nine. The results of the molecular docking are then visualized using UCSF Chimera [56]. In general, the predicted binding poses are able to fit well into the pocket when compared to the experimentally determined binding poses (Figure 7B). This demonstrates the utility of integrating AutoDock Vina into the workflow for the purpose of predicting the binding poses of a ligand. The binding affinity values for the predicted conformations on the sPARbs of 5EL7, 5IQR, and 5NDV are quite similar to each other (−8.4, −8.8, −9.0, and −8.0 kcal/mol, respectively; Figure 7B). The similarity in binding affinity values is consistent with the fact that all those binding sites are known to bind paromomycin.

### 3.4. Application of 3D Base Arrangement Comparisons to Identify Pseudoknots

The largest motifs that has been annotated by the NASSAM webserver are kink-turn motifs [22]. Its capability can be extended to annotate even larger motifs, including pseudoknots. Such motifs are found in various catalytically active RNA molecules, such as ribozymes [60], self-splicing introns [61], and telomerases [62]. They are also found in non-catalytically active RNA molecules, such as mRNA, that are involved in programmed −1 ribosomal frameshifting [63,64,65]. Due to its importance, there are already several programs that have the capability to annotate such pseudoknot motifs [14,34,66].

**Figure 8:**
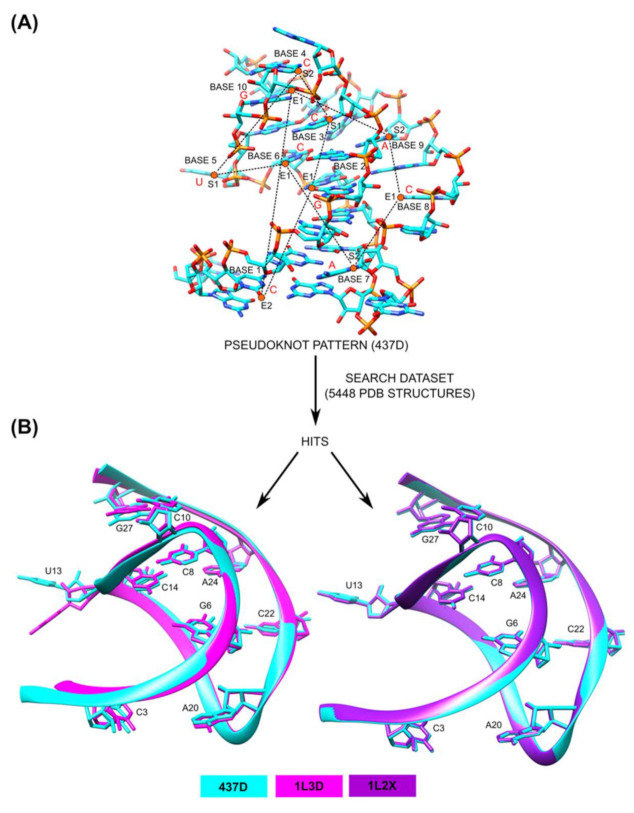
Annotation of pseudoknot motifs in RNA structures from PDB by NASSAM. (**A**) The 28 nucleotides (components) of a pseudoknot motif from the beet western yellow virus (PDB ID: 437D). The components selected to construct the pattern are labeled as Base 1 to Base 10. The red-colored letters indicate component types, either A, G, U, or C. The pseudo-atoms of the pattern are denoted as graph nodes (orange circles), and the distances between pseudo-atoms are denoted as graph edges (black dashed lines). Only a few of the pseudo-atoms and their distances are shown for clarity. (**B**) The pattern (PDB ID: 437D) base arrangements, shown as cyan-colored stick structures, are superimposed to the base arrangements of hits (PDB ID: 1L3D, 1L2X) shown as magenta-colored and purple-colored stick structures, respectively. The bases are labeled based on the sequence identifiers of the hit structures. The superimpositions of the backbones between the pattern and the hits are also shown to illustrate that the hits adopt pseudoknot structures.

The RNA pseudoknot from the beet western yellow virus (PDB ID: 437D) [64] was used to generate the pattern for a pseudoknot motif that was used as a query for a motif search by the NASSAM program. The pseudoknot motif from 437D consists of 28 nucleotides; however, only ten were selected for the vectors to construct the motif for use with a NASSAM search (Figure 8A). The motif search by NASSAM was performed on a dataset of 5448 RNA chains containing PDB structures. The use of a redundant structure dataset was intentional as the other structures that contain the pseudoknot motifs with exact components are considered redundant structures of 437D based on the latest Representative Sets of RNA 3D Structures (version 3.189) [54] at the time of writing (Figure 8A).

The motif search by the NASSAM program at 40% distance tolerance was able to retrieve all redundant structures of 437D (PDB ID: 1L3D, 1L2X) [63] that contain the pseudoknot motifs without any false positives (Figure 8B). The superposition of the backbones of the pseudoknot motifs (ribbon structures) using UCSF Chimera [56] clearly shows the pseudoknots (Figure 8B). This demonstrates that when provided with suitable vectors, NASSAM is capable of retrieving larger arrangements that include quadruples and quintuples that extend beyond the triples originally associated with the NASSAM algorithm.

### 3.5. A Database of RNA Base Interactions

Although the NASSAM and COGNAC tools are available for use over the web for the annotations of specific user-provided structures of interest, they can also be used to carry out a high-throughput automated annotation of the available PDB structures that contain RNA chains. As a result, both had been integrated to carry out a whole PDB annotation for the tertiary arrangements in the NASSAM database and also for networks of bases interconnected by hydrogen bonds that are composed of two to six base components. The resolution cut-off for the structures annotated by InterRNA is 4.0 Å to ensure the reliability of the annotations. The result of this annotation process has been made available as a web-accessible InterRNA database—http://mfrlab.org/interrna/ (accessed on 1 August 2021) [31].

## 4. Conclusions and Future Directions

Expert manual visual curation of RNA structures has proven to be an important starting point that formed the foundations for our current understanding of RNA structure and function. Even for large and complex structures such as the ribosomal subunits, the contributions of a specialist curator have allowed the discovery of many novel motifs. However, as the volume of structures increase, manual examination and comparisons of the structures to discern atomic level differences are no longer practical. The emergence of computational annotation tools has, since then, allowed the discovery of novel motifs in addition to allowing the high-throughput and exhaustive curation of tertiary sub-structures, which was not possible merely via molecular-graphics-aided visual examinations. The past two decades have seen significant progress made in the development and deployment of RNA 3D structure annotation applications. Nevertheless, there is a clear need for the adoption of standardized formats and nomenclature that would enable the outputs of the various tools to better interact and allow for cross-referencing in the near future.

## Figures and Tables

**Table 1:** Comparison of programs that annotate RNA base motifs in terms of the methods employed, inputs, availability, and motifs annotated.

**Programs**	**MC-Annotate**	**NCIR**	**RNAView**	**SCOR**	**DIAL**	**RNAJunction**	**R3D Align**	**RNA Frabase 2.0**	**WebFR3D**	**Nassam Webserver**	**RNA Base Triple Database**
Method	Structural graph	Literature survey	Coordinate frame as reference and least-square fit	Manual curation	3D-structure alignment	Predicts helices and determines their connectivity	Local alignment graph	Matching pattern in dot-bracket format	Geometric or symbolic matching (FR3D)	Sub-graph matching of vector arrangements	Symbolic matching (FR3D)
Input	3D structure	N.A.	3D structure	N.A.	3D structure	Sequences or PDB structure identifiers	3D structure	Sequence or secondary structure	Symbols (up to 15 nucleotides) or PDB structure identifiers	3D structure	N.A.
Availability	Webserver	Database	Webserver	Database	Webserver	Database	Webserver	Database	Webserver	Webserver	Database
Motifs annotated	Base pairs, Base triples, U-turn	Base pairs, base triples, base quadruples	Base pairs	Ribose zippers, T-loops, A-minor, pseudoknots, tetraloops	No specific motifs	RNA junctions, kissing loops	No specific motifs	Base pairs, base triples, base quadruples, base quintuples, hairpin loops, internal loops, junctions	GNRA loops, T-loops, sarcin-ricin loops, kissing loops, C-loops, A-minor, kink-turn	Base pairs, base triples, A-minor, T-loop, ribose-zippers, kink-turn, tetraloops	Base triples
**Programs**	**RNA 3D Motif Atlas**	**COGNAC Webserver**	**ClaRNA**	**RNA Bricks**	**RNAMotifScanX**	**InterRNA**	**CompAnnotate**	**RNApdbee 2.0**	**Local STAR3D**	**DSSR with PyMOL**	**ElTetrado**
Method	3D-structure alignment (FR3D)	Sub-graph matching of connection table graph representation	Geometric matching to reference dataset of ribonucleotide doublet	Secondary structure graph and superposition of 3D motifs to query structures	Alignment of interaction graph	Sub-graph matching using NASSAM and COGNAC	3D-structure alignment and comparative geometric assessments using high-resolution reference	Annotation of secondary structures to predict 3D interactions	Local com-patible graph alignment	DSSR geometric algorithm	Categorize quadruplexes based on secondary structure topology and component tetrads
Input	N.A.	3D structure	3D structure	3D structure	3D structure	N.A.	3D structure and base-pairing annotations	3D structure	3D structure	3D structure	3D structure
Availability	Database	Webserver	Webserver	Database	Offline executable	Database	Offline executable	Webserver	Offline executable	Webserver, Plugin in PyMOL	Offline executable
Motifs annotated	Sarcin-ricin loops, GNRA loops, T-loops, kink-turn, C-loop	Base pairs, base triples, base quadruples, base quintuples, base sextuples	Base pairs,base stacking, base-phosphate, base-ribose	Loops, stems, single-stranded	Kink-turn, C-loop, sarcin-ricin loops, reverse kink-turn, E-loop	Base pair, base interaction (triples-sextuples), ribose-zippers, A/G-minor motifs, hairpin loop, internal loop	Base pairs, kink-turn, C-loop, sarcin-ricin loops	Base pairs, loops, stems, single-stranded, quadruplexes, pseudoknots	No specific motifs	Base pairs, kissing loops, junctions	quadruplexes

## Data Availability

PDB: https://www.rcsb.org, InterRNA: http://mfrlab.org/interrna/.
